# Rodent-borne zoonoses in Qatar: A possible One-Health framework for the intervention of future epidemic

**DOI:** 10.1016/j.onehlt.2023.100517

**Published:** 2023-02-26

**Authors:** Md. Mazharul Islam, Elmoubashar Farag, Mohammad Mahmudul Hassan, Syed Shariq Jaffrey, Muzzamil Atta, Abdulla M. Al-Marri, Abdulaziz M. Al-Zeyara, Hamad Al Romaihi, Devendra Bansal, Zilungile L. Mkhize-Kwitshana

**Affiliations:** a Department of Animal Resources, Ministry of Municipality, Doha, Qatar; b School of Laboratory Medicine and Medical Sciences, College of Health Sciences, University of KwaZulu Natal, Durban 4000, South Africa; c Ministry of Public Health, Doha, Qatar; d Faculty of Veterinary Medicine, Chattogram Veterinary and Animal Sciences University, Chattogram 4225, Bangladesh; e Queensland Alliance for One Health Sciences, School of Veterinary Science, The University of Queensland, QLD 4343, Australia; f College of Animal Production, Bahry University, Khartum, Sudan; g Division of Research Capacity Development, Medical Research Council, Tygerberg, Cape Town 7505, South Africa

**Keywords:** One Health, Qatar, Rodent, Rodent-borne zoonoses

## Abstract

The increasing frequency of spillover of zoonotic pathogens from animals to humans in recent years highlights a need to develop a more comprehensive framework to investigate and prevent pathogens of animal origin, including rodents. Despite the presence of several species of rodents, there is a certain knowledge gap regarding rodent-borne zoonoses in Qatar. The current review provides an update on rodent-borne zoonoses in Qatar, its possible drivers and transmission dynamics, and proposed a One Health framework for intervention. Following an extensive literature review, we conducted a field investigation. Then the qualitative information and knowledge gaps were addressed with a virtual discussion with national, regional, and international experts in the relevant field. Overall, *Rattus norvegicus* population was found to be more prevalent, followed by *Rattus rattus*, and *M. musculus*, which are mainly found in animal farms, followed by agricultural farms, residential areas, and other facilities. Over 50% of rodents carry at least one pathogen of public health importance. Several pathogens were identified at the human, animal, and ecosystem interface, which can be mediated in transmission by rodents. *E. coli*, *Salmonella* spp.*,* and *Campylobacter* spp. are the frequently reported bacteria. *Hymenolepis* spp., *Cryptosporidium* spp., *Giardia* spp.*, Entamoeba* spp., and *Toxoplasma* spp. are the major parasites. In addition, many vectors, including *Ornithonyssus bacoti* and *Xenopsylla astia* were reported in this country. Based on the changes over the past 70 years in Qatar, seven drivers have been identified, which could be important in rodent-borne disease emergences, such as the Oil and gas revolution, fast population growth, rapid urbanization, importation of food and agricultural products, agricultural and livestock development, farm biosecurity, and stray animals. The experts emphasized that mixed-species animal farming with poor biosecurity and management can be associated to increase the risk of zoonoses. Moreover, rapid urbanization and global climate change together can alter the ecosystem of the country and impact on vectors and vector-borne diseases. Finally, the One Health framework has been proposed for the surveillance, and mitigation of any future spillover or epidemic of rodent-borne zoonoses.

## 1 Background

Rodents are an essential component of every terrestrial ecosystem. With about 36 families and 2552 species, these animals are the most diverse and highest representing (40% of the total mammals) in the mammalian world. These animals have high evolution abilities, such as high prolificacy, short gestation period, robust body shape, and small to medium-sized bodies that acclimatized them for surviving in different ecosystems [1], including deserts. They serve to land habitat ecosystems by modification of the soil structure, increasing soil aeration and hydration, energy and nutrition cycling, seed and spore pollination and dispersal, and source of feed for other animals (eagle, fox, and cat) [1]. In addition, they cause significant harm, such as consuming, contaminating, and damaging our foods, destroying property (clothes and documents) and structure (floor and networking facilities), and sometimes causing accidents (damage to the electrical network) [2]. These animals also have serious concern of transmitting pathogens of public health importance [3,4], and facilitating the transmission of at least 84 zoonotic diseases as carriers or reservoirs [5]. The commensal rodents have higher public health significance as they carry pathogens directly to humans and other animals (livestock and pets) through contact with rodent feces, urine, and skin/fur and indirectly through ectoparasites and other vectors [6]. There are several histories of rodent-borne zoonotic disease epidemics globally, such as plague, leishmaniasis, and typhus fever [[7], [8], [9], [10]]. In recent years, the increasing frequency of spillover of zoonotic pathogens from animals highlights a need to develop a framework to investigate and prevent pathogens from rodents.

Qatar is a small peninsular Arab country, home to people from over 90 countries, most of whom are from South Asia. There are four species of rodents reported in this country, one is the wild species: *Jaculus jaculus* [11], and others are commensal species: *Mus musculus, Rattus norvegicus, and Rattus rattus* [[11], [12], [13], [14]]. To prevent any rodent-related zoonoses epidemic in the future in this country, especially during the FIFA World Cup 2022, a study was conducted on commensal rodents and rodent-borne pathogens. Several articles were published based on this research [5,[15], [16], [17], [18], [19]], which suggest that rodent-borne zoonoses is not a neglected issue. Therefore, intervention on such zoonoses needs to be emphasized. One Health is a multisectoral collaborative approach, which is an essential component in combatting health issues at the human-animal-ecosystem interface. Applying this approach in prevention and control of zoonotic diseases can save lives, which also ensures an efficient use of resources (fund, facility, and personnel), and a quality healthcare delivery in a timely manner. Despite increasing success and awareness of the One Health approach, shortage of communication, coordination and structured guideline for the relevant sectors is still hindering implementation of this tactic [20]. The current review provides an update of rodent-borne zoonoses in Qatar, as well as possible drivers and transmission dynamics of such zoonoses. The present study also proposes a possible One Health intervention program to prevent such zoonoses epidemics in this country.

## 2 Materials and methods

### 2.1 Literature review

Before the start of the review itself, the authors shared their knowledge and views with each other using Delphi Method to understand the rodent-borne zoonotic pathogens, their drivers, and transmission dynamics at Qatari ecosystem and culture. Then we conducted an extensive literature review for relevant articles published between 2000 and middle of 2022 in Google scholar, PubMed, Science Direct, Web of Science, and Scopus in terms of rodent-borne bacteria, virus, helminths, protozoa, and vectors at the human, animal, and ecosystem interface of Qatar [5,15,16]. We used Boolean operations for the key words to enhance retrieval of relevant articles and narrow the search results. In addition, references of the selected articles were examined to cover the related articles. We also conducted electronic and hand search for the grey literature related to Qatar population, economy, trade, and development from different ministries of Qatar, including the Ministry of Public Health, Ministry of Municipality and Environment, and Qatar Statistical Authority, and international organizations, such as the World Bank, World Health Organization, and World Organization for Animal Health. The associated citizen science documents were studied in the blog posts, social media, and newspapers.

### 2.2 Field investigation

We conducted a cross sectional study starting from the year 2018 to 2019 to assess presence of rodent-borne zoonotic pathogens in Qatar. As such, we trapped 148 rodents from some selected areas of the country and conducted gross external examination, necropsy, coproscopy, histopathology, bacterial culture and identification, ELISA, PCR, and sequencing to understand the zoonotic pathogens. Detail of the rodents and their related pathogens were described previously [[17], [18], [19]].

### 2.3 Expert panel discussion

Subsequently, a One Health expert panel was held in September 2022 via Microsoft Teams to share information and address remaining knowledge gaps. The panel was distributed into two sections based on the availability of the experts who had been invited. A group of 27 experts attended the first meeting, whereas 14 experts were in the second session. The experts were from national, regional, and international level in the fields of animal health, public health, or environment department. Criteria for expert selection included: 10 years or more experience to work in public health, animal health, zoonotic disease, livestock or agricultural farm management, rodent control, rodent disease research, national or international policy making in public health. Additionally, minimum of 10 years residential experience within Qatar context was also required. Both meetings were conducted in English language and lasted for at least one hour. One of the authors was assigned to be a moderator and recorded the meetings while the rest were participants.

During the meetings, a brief presentation was shared with the experts which explained the research findings on rodent and rodent-borne zoonoses in Qatar. Afterwards, a list of challenges and knowledge gaps was shared with the experts. These gaps included: increased the risk of rodent-borne zoonoses in Qatar from imports of food and agricultural products, role of immigrant workers in transmission of these diseases inside Qatar and cross-border transmission, impact of climate change and urbanization in Qatar on rodent-borne disease transmission, management of rodents in traditional farming and old housing systems of the country, and major risks of rodent-borne disease within the country. Afterwards, the experts were asked to share possible interventions for the aforementioned challenges using One Health approach. Finally, the two meetings were transcribed and converted in a single document and shared with the experts for verification.

## 3 Results

### 3.1 Overview of rodent-borne pathogens

Overall, *R. norvegicus* found to be more prevalent followed by *R. rattus*, and *M. musculus* in Qatar [17] and mainly distributed in the animal farms, followed by agricultural farms, residential areas, and other facilities (commercial and industrial areas). Over 50% of rodents of Qatar carry at least one pathogen of public health importance [19].

#### 3.1.1 Bacterial pathogens

A total of 23 bacterial species were reported at the human, animal, and environment interface in Qatar (Table 1). The majority of them were found in humans and rodents. *Campylobacter* spp.*, Escherichia* spp., *Salmonella* spp. and *Rickettsia* spp. were reported in domesticated animals, such as livestock, poultry, and pets. In addition, *Campylobacter* spp.*, Corynebacterium* spp.*,* and *Escherichia* spp. were notified from the environment samples of the animal farms and live animal markets.

**Table 1:** Rodent-borne zoonotic bacteria reported in Qatar.

Bacteria names	Reporting hosts	References
*Acinetobacter baumannii*	Human and Rodent	[18,21]
*Aeromonas salmonicida*	Rodent	[18]
*Campylobacter jejuni*	Chicken, camel, cattle, sheep, Human, Animal product and farm	[[22], [23], [24]]
*Campylobacter coli*	Chicken, camel, cattle, sheep, Human, Animal product and farm	[[22], [23], [24]]
*Campylobacter laridis*	Human	[24]
*Campylobacter upsaliensis*	Human	[24]
*Corynebacterium* sp.	Central fresh product market	[25]
*Citrobacter freundii*	Rodent, Human	[18,26]
*Citrobacter koseri*	Rodent, Human	[18,26]
*Coxiella burnetii*	Human	[27]
*Enterobacter aerogenes*	Rodent, Human	[18,26]
*Enterobacter cloacae*	Rodent, Human	[18,26]
*Escherichia coli*	Human, camel, cattle, chicken, and sheep, rodent, and environmental samples	[18,25,[28], [29], [30], [31], [32], [33], [34], [35], [36], [37]]
*Hafnia alvei*	Rodent	[18]
*Klebsiella pneumoniae*	Rodent, Human	[18,26]
*Listeria monocytogenes*	Human	[38]
*Providencia stuartii*	Rodent	[18]
*Proteus mirabilis*	Rodent	[18]
*Pseudomonas aeruginosa*	Rodent, Human	[18,26]
*Mycobacterium tuberculosis*	Human	[[39], [40], [41], [42], [43]]
*Rickettsia* spp.	Rodents, dogs, and cats	[18,44,45]
*Salmonella* spp. (including *Salmonella enterica*)	Animal holding, human, livestock (camel and cattle), and rodents	[18,24,[46], [47], [48], [49], [50], [51], [52]]
*Yersinia* spp.	Human	[34]

#### 3.1.2 Viral pathogens

Rodents can be involved in transmission of chikungunya and hepatitis E, which have been reported among humans (Table 2). Additionally, rabies was identified in both humans and animals. Evidence of seroconversion to WNV-pE-Ab were detected in horses of Qatar [58].

**Table 2:** Rodent-borne zoonotic viruses and viral antibodies reported in Qatar.

Pathogen name	Reporting hosts	References
Chikungunya virus	Human	[53]
Hepatitis E virus	Human	[54,55]
Rabies virus	Human, camel, and fox	[56]
West Nile Disease virus	Human, horse	[57,58]

#### 3.1.3 Parasitic pathogens

Several rodent-borne parasites were reported in humans and animals of Qatar (Table 3). *Hymenolepis diminuta* has been reported to be a highly prevalent helminth in rodents in this country. Whereas *H. nana* was found only in humans. *Toxoplasma gondii* is prevalent in humans, rodents, and cats. *Leishmania* spp. was found in dogs and cats.

**Table 3:** Rodent-borne zoonotic parasites and antibodies to the parasites reported in Qatar.

Parasite name	Reporting hosts	References
Helminths		
*Echinococcus granulosus*	Humans, sheep, and goats	[59]
*Hymenolepis diminuta*	Rodents	[19,22]
*Hymenolepis nana*	Humans	[60,61]
*Schistosoma mansoni*	Humans	[62,63]
*Taenia* spp.	Humans	[61,64]
*Taenia taeniaeformis*	Cats and rodents	[19,65,66]
*Toxascaris leonina*	Cats	[65,67]
*Trichuris trichiura*	Humans	[68,69]

Protozoa		
*Babesia* spp.	Cats, dogs, and humans	[[45], [70]]
*Cryptosporidium* spp. (*C, parvum, C. hominis, C. meleagridis*)	Humans	[71,72]
*Entamoeba* spp. (*E. hominis, E. disper*)	Humans	[61,71,73]
*Giardia* spp. (*G. duodenalis*)	Humans and rodents	[19,61,73]
*Leishmania* spp.	Cats, dogs, and rodents	[19,74]
*Toxoplasma gondii*	Cat, rodents, and humans	[19,[75], [76], [77], [78]]
*Trypanosoma lewisi*	Rodents	[19]

### 3.2 Factors associated with rodent-borne pathogens

During the review of the literature and the field investigation, we identified several factors, which can be associated with rodent-borne pathogen emergence in Qatar.

#### 3.2.1 Vectors

Many vectors, which can facilitate in transmission of several bacterial, viral, and parasitic diseases were found in Qatar (Table 4). *Ornithonyssus bacoti* and *Xenopsylla astia*, which can be the vectors of *Rickettsia* spp. were reported in rodents. In addition, several other vectors, such as sand fly, ticks, mosquito vectors are available in this country.

**Table 4:** Public health importance vectors reported in Qatar that can intervene rodent-borne zoonoses transmission.

Vector	Reported hosts	Potential role for pathogen transmission	References
Sand fly			
*Phlebotomus papatasi*	–	*Leishmania* spp.	[79,80]

Flea			
*Xenopsylla astia*	Rodent	*Yersinia pesits, Rickettsia* spp., *Hymenolepis diminuta*	[14,80]
*Xenopsylla cheopis*	Rodent	*Yersinia pestis, Hymenolepis diminuta, Rickettsia* spp., *Bartonella* spp.	[[79], [80], [81], [82], [83], [84]]
*Ctenocephalides felis*	Cat	*Bartonella* spp. *Rickettsia* spp.	[79,80]

Mite			
*Ornithynyssus bacoti*	Rodent	*Coxiella burnetii, Rickettsia* spp.	[19,81,82]

Ticks			
*Hyalomm dromedarii*	Camels, cattle	Crimean-Congo hemorrhagic fever virus, *Rickettsia* spp., *Coxiella burnetii*	[79,80]
*Hyaloma impeltatum*	Camels, cattle, sheep	Crimean-Congo hemorrhagic fever virus, *Rickettsia* spp.	[79,80]

Mosquito			
*Aedes aegypti*	Humans	Chikungunya virus	[79,80]
*Culex pipiens*	Human	West Nile Virus	[79,85,86]
*Culex univittatus*	Mammals	West Nile Virus	[79,80]

#### 3.2.2 Rodent control services

There are no clear rodent control guidelines in Qatar. Ministry of Municipality of Qatar has pest control unit, which provide public service in pest control to the citizens and residents. There are several corporate services which manage pest control within the country, including rodents. These pest control service providers work based on their individual strategies. The service beneficiaries are mostly corporate offices, industries, residential areas, major public parks, and other public places. However, limited interventions are applied in livestock and agricultural farms.

#### 3.2.3 Drivers of rodent-borne zoonoses

Based on the changes over the past 70 years in Qatar, some of the following drivers could influence rodent population and rodent-borne disease emergence in Qatar: (1) oil and gas revolution, (2) rapid increase of multicultural population, (3) rapid urbanization, (4) importation of food and agricultural products, (5) agricultural and livestock development, (6) farm biosecurity, and (7) stray animals.

**Fig. 1:**
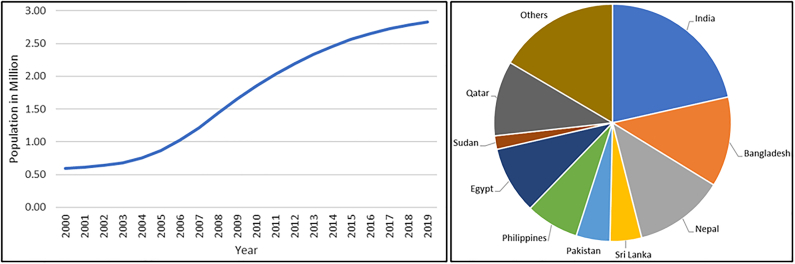
Qatar population growth from 2000 to 2019 (left) and population distribution by country of origin in 2019 (right) [91,92,97].

In the past, Qatar was inhabited primarily by Bedouins who mainly delved in fishing, pearl harvesting, and livestock farming. Ever since the oil revolution in the 1950s, economic development and globalization [[87], [88], [89]] has attracted high numbers of skilled, semi-skilled, and un-skilled workers [88,90] (Fig. 1). This migration played an important role in determining population change and a dynamic socio-economic environment in this country. In 1960s, Qatar's population was only 47,000, by the 21st century it had increased 12 folds [91], with over 2.7 million in 2020 with considerable industrial and commercial development [92]. This change could disrupt the rodent ecosystem in the deserts, forcing them to migrate toward the cities. Generally, rodents tend to live within city limits due to vast availability of food resources and less risk of predation [93].

**Fig. 2:**
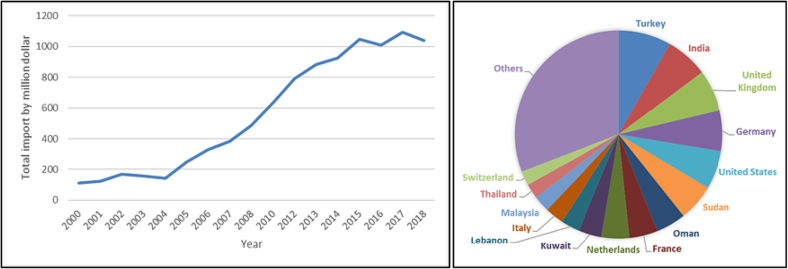
Food and agricultural product import in Qatar from 2000 to 2018 (left) and the partner countries to import in 2018 (right) [96,97].

As a desert country, Qatar initially had minimal agricultural activities but that has changed in the last decade [94,95]. The country imports food and agricultural products from different countries. The annual cost of food import rose from around $100 million in 2000 to $1000 million in 2018 [96] (Fig. 2). Some major partner countries for importation of manpower, live animal and food products include: India, Bangladesh, Nepal, Sri Lanka, Pakistan, Philippines, Egypt, Turkey, and Sudan [96,97]. These countries are endemic with many rodent-related diseases, such as rabies, typhoid fever, chikungunya, leishmaniasis, and Rift Valley fever [[98], [99], [100], [101], [102]]. As such, any kind of trade or travel from these countries presents a risk of various diseases being imported within Qatar [5].

**Fig. 3:**
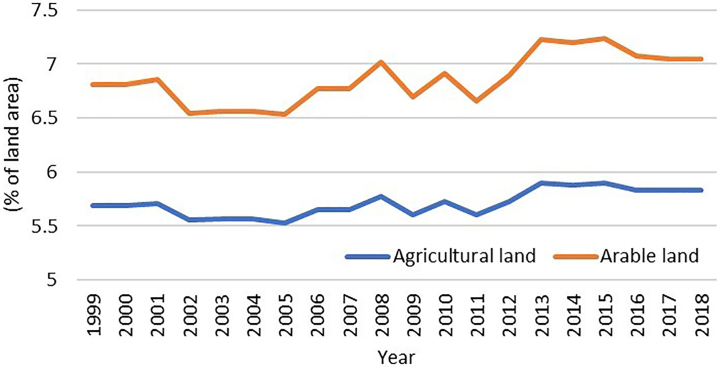
Changes in land use for agricultural and arable purpose from 1999 to 2018 in Qatar [96].

**Fig. 4:**
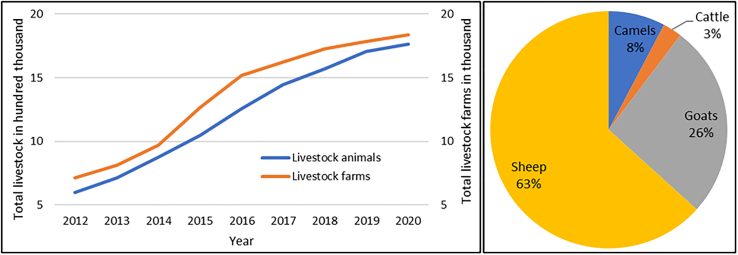
Changes in number of total livestock and livestock farms from 2012 to 2020 (left) and distribution of livestock animals in 2020 (right) [103].

There has been an increase in agricultural practices in recent years in Qatar. The amount of vegetated area and arable land increased from 5.3% to 5.8%, and 6.8% to 7.2%, respectively from 1999 to 2018 [96] (Fig. 3). Since the blockade in Qatar in 2017, the local food production has increased, and several animal and vegetable farms started operating locally to distribute food in the market for human consumption. The number of livestock and livestock farms has also been increased by 3 times in the last 10 years [103] (Fig. 4). In Qatar, livestock farms are mainly managed traditionally with insufficient biosecurity measures. These farms are generally keeping mixed livestock, captive wild animals, exotic birds, and pets within a single boundary (usually within 50 × 50 square meter space). The majority of farmworkers are from Bangladesh, India, Nepal, and Sudan and live in the farm compound. The owners keep their resting place (majlis) inside the farm where they spend their evening and holiday times. Our study demonstrates that livestock and agricultural farms are more prevalent with rodents than other areas [17]. Due to the close proximity between humans and multispecies animals with poor biosecurity management in the Qatari livestock farms, there is the chance of species jumping of zoonotic pathogens at human and animal interface, observed in MERS-CoV cases. The owner, worker, and camels of the same farm were found positive for MERS-CoV [87]. Although there is no documented report of stray dogs and cats in Qatar, according to the newspaper reports [[104], [105], [106]], there is a rise in the numbers of stray cats and dogs in this country, which can influence the transmission cycle of the rodent-borne pathogens [5].

#### 3.2.4 Possible transmission dynamics

**Fig. 5:**
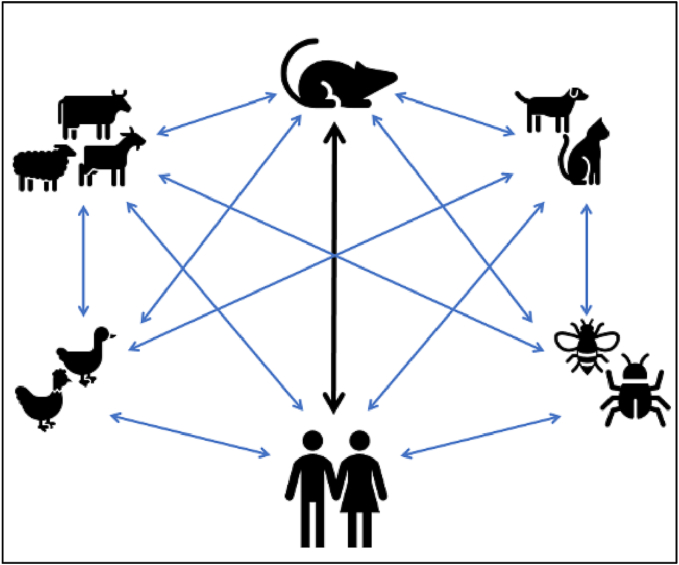
Possible transmission dynamics of rodent-borne zoonotic diseases in Qatar.

**Fig. 6:**
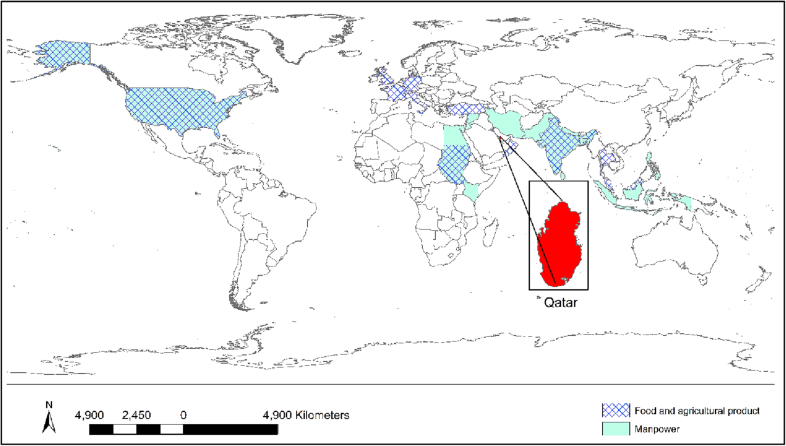
The global map showing the top 15 partner countries for manpower, food, and agricultural product importation. Transboundary transmission of such diseases can happen to Qatar from these partner countries.

Rodent-borne pathogens can be transmitted to humans by direct contact from rodents, however, they can also be mediated through inter and intra-species transmission among vectors or reservoirs, such as livestock, pets, and arthropods (Fig. 5). Certain diseases can be imported to Qatar by the immigrant workforce, pets, and food and agricultural product importation, such as Leishmaniasis, Salmonellosis, Mpox, and Rabies (Fig. 6). Many partner countries, such as Bangladesh, India, Nepal, Sri Lanka, Iran, Pakistan, and Egypt from where major immigrant work force, food and agricultural products imported are endemic with rodent-borne zoonotic diseases, such as, *Hymenolepis nana*, Hepatitis E, Enteric fever, Leishmaniasis, and Schistosomiasis [51,52,[107], [108], [109], [110]]. Most of these diseases were reported among the immigrant residents in Qatar, especially among the newly immigrant residents, which indicates that such diseases were imported to Qatar by the immigrant workforce, such as Giardiasis and Cryptosporidiasis [61,73,111].

### 3.3 Expert opinion

The species diversity, population density and distribution, and population burden of rodents in Qatar is still unknown. Which is why it is essential to conduct scientific investigations to estimate actual rodent population density and geographic distribution, especially to identify abnormal population diversity, habitats, and ecology. The experts' opinion emphasized that mixed species animal farming, inadequate biosecurity measures, and poor animal housing and agricultural practices in farms tend to increase the risk of zoonoses. Urine and feces from rodents can contaminate animal feed, which can eventually lead to transmission among animal handlers, especially if there is a lack of biosafety and biosecurity measures implemented on the farms. Development and implementation of appropriate legislation and guidelines can be used to mitigate such risks and ensure placement of biosafety and biosecurity.

Rapid urbanization and global climate change together can alter the ecosystem of a country and directly impact proliferation of vectors and vector-borne diseases (VBDs), especially with regards to change in pathogen characteristics, host diversity, transmission, and distribution, finally emergence of novel pathogens. It was highly emphasized that vectors need to be investigated to check for the presence of pathogens to mitigate any possible threat of VBDs as early as possible. It was mentioned that dogs and falcons should also studied since they are used in sporting activities and can possibly contract VBDs, then transmit them through contact with infected rodents. By extension, stray cats too should be kept in check in order to avoid spread of VBDs. Some experts cited Oman, a member country of the Arabian Peninsula, as an example of emergence for Schistosomiasis with immigrant workers, while some others mentioned the risk of a pathogen being imported by immigrant workers is minimal, even via food imports. Overall, they all agreed that risk of imported pathogen should not be omitted without proper investigation. Consensus between the experts was to conduct a risk assessment of rodent-borne zoonoses to gauge existing gaps and mitigate these risks. Additionally, seaports and airports should be focus of stronger surveillance practices to minimize risks of rodent-borne zoonoses in Qatar. Regarding food chain, experts think that it is essential to take into consideration eating preferences of those coming in from abroad, as people from some countries, e.g., tribal peoples of Nepal, India, and Thailand commonly eat rodents.

It was mentioned that Qatar's population, including citizens and residents should be monitored by ranking them into two categories, high risk and low risk with focus on their work and accommodations. Immigrant workers, more broadly, people living in socio-environmentally degraded areas, such as agricultural and livestock farms and old construction housing facilities would be at high risk of infection. It is equally important to involve residents of the country using citizen science for surveillance related activities. An electronic database accessible through mobile application can be used by citizens to send relevant information which can then be retrieved by health professionals. Furthermore, experts mentioned that it would be prudent to institute knowledge, attitudes, and perception studies to understand the situation from the perspective of the citizens through which we can gauge current gaps and address them as such. It was also highly advised that the municipality notification system for inspection and control can be extended to study zoonoses. Finally, the experts suggested to conduct the surveillance under One Health approach with multidisciplinary expertise team.

## 4 Discussion

The prevalence of rodent-borne pathogens is a complex phenomenon as it has a consequence of a combination of many factors, which includes presence of rodent population, availability of intermediate hosts such as vectors, trapping method and location (cleanliness, use of rodenticide, insecticide, and management), and climatic situation (temperature, humidity, and rainfall). The current study shows that rodents in Qatar are important for zoonotic pathogens and relate vectors. Rodent population index is still unknown in this country, although the commensal rodents are mostly distributed in the livestock farms [17]. In general, 10% of these animals among their total population in an ecosystem act as pests, where it is five time more in Qatar [1,112]. In addition, presence of several rodent-borne pathogens in humans, other animals, and in the environmental samples increase the risk of zoonoses transmission at the One Health interface. Although, experts suggest that importation of a pathogen through food and agricultural products and immigrant workers from the endemic countries is minimal, however, the risk cannot be eliminated or ignored. Further extensive investigation is required to understand pathogen importation and dissemination dynamics to formulate effective action plans to prevent any future spillover of these pathogens. The goal should be to reduce possible rodent overpopulation and institute early preparedness measures. Outbreak investigation and early detection of the pathogens using a multidisciplinary team is essential.

It is important to develop a surveillance system to explore rodent demography and population saturation, and create epidemiological profile of rodent-borne zoonoses in Qatar. Working with rodents needs time, money, dedication, facilities, and expert involvement. In rodent-borne disease surveillance, a single/specific disease surveillance is not practical. The traditional segmented approach will only increase cost consumption and time requirements, whereas joint-team work can enhance sharing of knowledge via lateral multisectoral collaboration for maximum efficiency. Capacity development for pathogen detection by both medical and veterinary sectors was highly suggested to respond and manage rodent-borne zoonotic threats at the earliest interval possible. Therefore, it might be helpful to prioritize rodent-borne diseases, which will not necessarily need the understanding of the whole ecology but only the common drivers and risks based on the context that will alleviate the burden from control program. A roadmap identifying priorities for research and development, and capacity building in rodent control programs is required for a systematic execution of action plan. In this regard, One Health approach has shown success in cross-sectoral collaborative exercises for emerging disease research, such as outbreak investigation, surveillance, early detection of pathogens in many countries [113,114], including Qatar [115]. The “National Outbreak Control Task Force” and One Health framework [116] of Qatar can be strengthened through capacity-building, formulating supportive legislation, allocating budget, and engaging relevant international and national organizations. It also should follow the International Health Regulation (2005) guidelines [117].

**Fig. 7:**
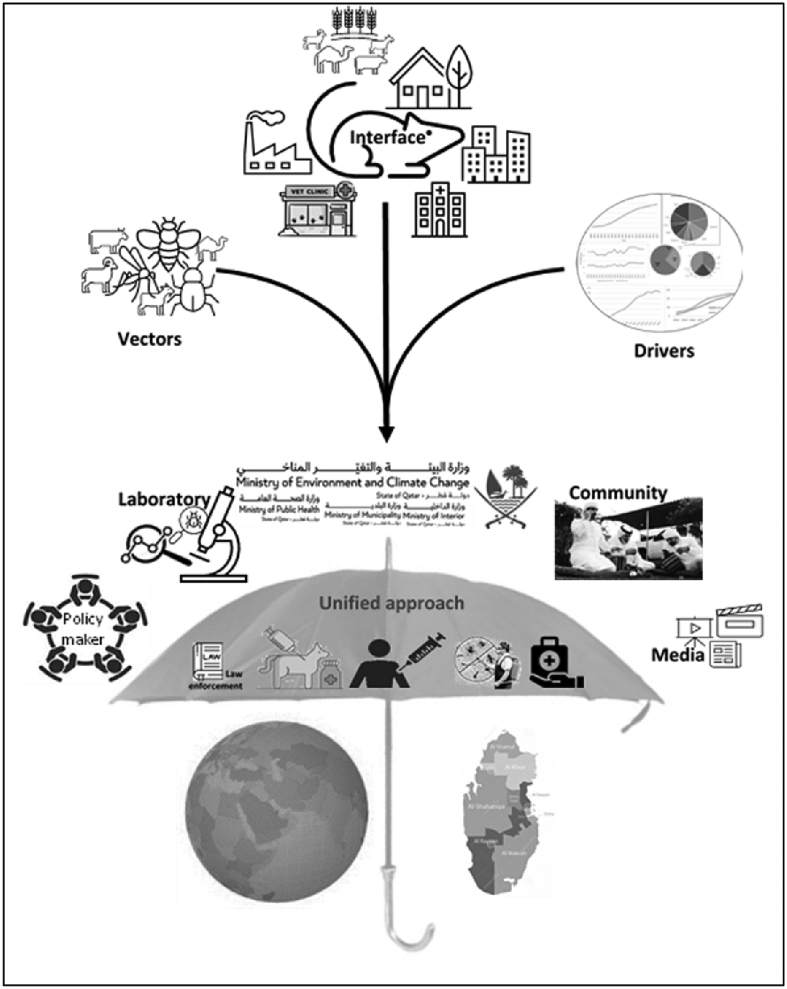
Evidence based One Health investigation to prevent rodent-borne zoonoses. Rodent infestation reports will be provided to the One Health team. The One Health team will collect the records of vectors and hospitals and analyze with rodent infestation. Then after joining the local community, policy makers, media and lobby groups, a unified decision will be taken and applied to investigate and prevent rodent-borne zoonoses in Qatar.

The concept of One Health advocates for a holistic approach to tackling diseases at the human-animal-ecosystem interface. Rodent-borne zoonoses are the classic example of where the One Health movement can play a key role in stifling their spread. Thusly, multisectoral engagement is needed from several stakeholders, including rodentology, veterinary, medical, ecology, policy makers, lobby groups, and media; considering the local socio-economic, cultural and spiritual facets; and access of up-to-date technologies for pathogen surveillance and characterization; risk mapping, stratification, and prediction model preparation; translating research findings to disease control; and unified decision making. The rodent-borne disease prevention and control decision must include legislation, law enforcement, national pest control policy development, animal, and human vaccination (including rodent vaccination), farm biosecurity, infection control and prevention in the hospital premises, treatment to the relevant hosts, border control strategy. We propose a possible One Health framework for rodent-borne pathogen surveillance and control to combat future epidemics in Qatar (Table 5, Fig. 7).

**Table 5:** Possible One Health framework for rodent-borne pathogen surveillance and control to combat future epidemics in Qatar.

Risk assessment	One Health surveillance and pathogen control program		
Risks	Goals	Surveillance	Intervention
Rodents: • Four species of rodents: *Mus musculus, Rattus norvegicus*, *Rattus rattus*, and *Jaculus jaculus* Other animals: • Livestock farms keep multispecies animals at a close proximity Humans: • Multinational population, majority are from South Asia Environment: • Vectors, such as *Xenopsylla cheopis*, *X. astia*, *Ornithonyssus bacoti* are available Pathogens: • Risk of transboundary rodent-borne zoonoses entry with live animal trade, food, and agricultural products from endemic countries • 45 rodent-borne zoonotic pathogens (18 parasites, 23 bacteria, and 4 virus) • Epidemiology and transmission dynamics of the rodent-borne zoonoses are unknown • No national guideline for rodent and rodent-borne pathogen control • No national guideline for livestock and agricultural farm biosecurity practices	Detect: • Early detection of rodent-borne pathogen epidemics • Rodent population demography, habitat, abundance, and overload • Biology and epidemiology of the rodent-borne zoonoses, and risk mapping Prevent: • Reduce rodent overload • Prevent rodent infestation in the farms, residential, commercial, and industrial areas • Combat rodent-borne zoonoses Respond: • Outbreak investigation and surveillance for rodent-borne zoonoses • Early preparedness of any outbreak or epidemic • Rodent-borne zoonoses management	One Health team: • Multisectoral involvement, such as medical, veterinary, and environment specialists • The associated stakeholders from community, lobby groups, media, and policy makers Local capacity building: • Capacity building for surveillance and monitoring • Strengthen research collaboration • Disease modeling and translating research findings to field application Support of One Health movement: • Activate the National Outbreak Control Task Force of Qatar or strengthen it • Approve supportive legislation, timeline, and budget	• Integrated pest management policy development and implementation • Biosecurity policy development and implementation • Border control • Vaccination • Development of awareness • Rapid response • Consider the local socio-economic, cultural, and spiritual factors in One Health policy making

Rodent control is considered an important tool to prevent rodent-borne disturbances. The rodent control policy should go through three principles: (1) surveillance, monitoring, and evaluation; (2) set action thresholds; (3) control. Rodent and rodent-borne disease control and prevention is a public-private joint venture which requires a multistep and coordinated approach. It is not recommended to kill large populations of rodents. Infectious agents can remain even if 75% of rodents in a population is brought under control. Rodent-borne diseases can disappear after few years of continuous rodent control programs but can re-emerge if the control program is either stopped or rodent population increases. Even after the entire rodent population has been eliminated, the risk of pathogens can still persist as they can readily adapt to changes in the environment [93]. Since rodents are part of the local ecosystem, an ecological surveillance is needed to create a complete epidemiological profile of rodent demography, habitual distribution, associated factors for rodent abundance, and rodent overload in Qatar. Rodent survey and control should be organized under four major keywords: humanity, effectiveness, biosafety, and cost-efficiency. To accomplish this, it is essential to involve governmental authorities, engage the lobbyists, educate the citizens and residents, and essentially develop collective consciousness.

Although animal ethics are closely practiced for animals used in experiments, however, these principles are not followed for when certain animals are considered pests. Despite being regarded as pests, rodents deserve to be handled with humane considerations. Hence, an Integrated Pest Management (IPM) program can be amended within the rodent control program to ensure ethical measures are followed in management of rodents across Qatar [118]. A national IPM guideline can be developed, in which local facilities, ecosystem interaction between humans, livestock, pets, and rodents, socio-economic balance, local culture, and cost-benefit will be respected. IPM can serve as a quality check for biosafety and management procedures in the hospitals, residential complexes, business centers, airport, and seaports in Qatar. Different facilities require different length of control strategies to be put in place, such as short-medium-long. A short-term solution addresses the immediate rodent control problem, whereas a long-term plan focuses on understanding rodent ecology and risk factors. In the residential, corporate, and industrial areas, short-term or emergency rodent control is applicable. Higher emphasis should be given to the livestock and agricultural areas. Development and application of biosecurity measures in livestock and agricultural farms is vital in control of rodents and rodent-borne zoonoses. This can be achieved via support from legislation and law enforcement, which will not only help reduce rodent infestations, but also decrease prevalence of zoonotic diseases of concern.

## 5 Conclusion

This research provides an overview of rodents, rodent-borne pathogens, possible zoonoses, and risk of rodent-borne pathogens transmission in human-animal-environment interface in Qatar. Our understanding is that the proposed One Health framework will be a key tool for controlling or combating future spillover or epidemic in Qatar. Moreover, our proposed One Health Framework can be applicable in other countries to combat any future spillover or epidemic associated with rodents. Capacity development in conjunction with collaboration from different countries, who have had prior experience combating rodent and vector borne diseases, can be invaluable assets in rodent-borne disease control programs. Furthermore, a collective structured approach to surveillance and research priorities can achieved through collaboration with different ministries of Qatar, Qatar National Research Fund, Qatar University, Qatar foundation, and the pest control companies. Community awareness and engagement via local newspapers, radio, social media, and Qatar television, can also provide vital support needed to create an effective rodent-borne disease control program.

## Funding

No funding was received for this study.

## Declaration of Competing Interest

Authors declare no conflict of interest.

## Data Availability

Data will be made available on request.
